# Comparative Genomics Reveals New Candidate Genes Involved in Selenium Metabolism in Prokaryotes

**DOI:** 10.1093/gbe/evv022

**Published:** 2015-01-31

**Authors:** Jie Lin, Ting Peng, Liang Jiang, Jia-Zuan Ni, Qiong Liu, Luonan Chen, Yan Zhang

**Affiliations:** ^1^Key Laboratory of Systems Biology, Institute of Biochemistry and Cell Biology, Shanghai Institutes for Biological Sciences, Chinese Academy of Sciences, University of Chinese Academy of Sciences, Shanghai, China; ^2^Key Laboratory of Nutrition and Metabolism, Institute for Nutritional Sciences, Shanghai Institutes for Biological Sciences, Chinese Academy of Sciences, University of Chinese Academy of Sciences, Shanghai, China; ^3^Shenzhen Key Laboratory of Marine Biotechnology and Ecology, College of Life Sciences, Shenzhen University, Guangdong Province, China

**Keywords:** selenium metabolism, SelD, comparative genomics, prokaryotes

## Abstract

Selenium (Se) is an important micronutrient that mainly occurs in proteins in the form of selenocysteine and in tRNAs in the form of selenouridine. In the past 20 years, several genes involved in Se utilization have been characterized in both prokaryotes and eukaryotes. However, Se homeostasis and the associated regulatory network are not fully understood. In this study, we conducted comparative genomics and phylogenetic analyses to examine the occurrence of all known Se utilization traits in prokaryotes. Our results revealed a highly mosaic pattern of species that use Se (in different forms) in spite that most organisms do not use this element. Further investigation of genomic context of known Se-related genes in different organisms suggested novel candidate genes that may participate in Se metabolism in bacteria and/or archaea. Among them, a membrane protein, YedE, which contains ten transmembrane domains and shows distant similarity to a sulfur transporter, is exclusively found in Se-utilizing organisms, suggesting that it may be involved in Se transport. A LysR-like transcription factor subfamily might be important for the regulation of Sec biosynthesis and/or other Se-related genes. In addition, a small protein family DUF3343 is widespread in Se-utilizing organisms, which probably serves as an important chaperone for Se trafficking within the cells. Finally, we proposed a simple model of Se homeostasis based on our findings. Our study reveals new candidate genes involved in Se metabolism in prokaryotes and should be useful for a further understanding of the complex metabolism and the roles of Se in biology.

## Introduction

Selenium (Se) is an essential trace element in many organisms from bacteria to humans. The majority of biological effects of Se are exerted by selenocysteine (Sec, U), which is known as the 21st amino acid and is cotranslationally incorporated into selenoproteins by recoding the UGA codon from stop to Sec function (Bock et al. 1991; Stadtman 1996; Hatfield and Gladyshev 2002). The Sec biosynthesis and its insertion into proteins involve a complex machinery that includes both common core components and differences among the three domains of life (Low and Berry 1996; Driscoll and Copeland 2003; Yoshizawa and Bock 2009; Donovan and Copeland 2010).

To date, the mechanism of Sec insertion into proteins has been most thoroughly elucidated in selected groups of prokaryotes and eukaryotes (Bock et al. 1991; Low and Berry 1996; Stadtman 1996; Bock 2000; Hatfield and Gladyshev 2002; Thanbichler and Bock 2002; Driscoll and Copeland 2003; Allmang et al. 2009). In bacteria such as *Escherichia coli*, the biosynthesis and specific insertion of Sec require an in-frame UGA codon, a *cis*-acting Sec insertion sequence (SECIS) element which is a hairpin structure within the selenoprotein mRNA immediately downstream of the Sec-encoding UGA codon, and several *trans*-acting factors dedicated to Sec incorporation. Briefly, the SECIS element binds to a Sec-specific elongation factor SelB and then forms a complex with Sec-tRNA^[Ser]Sec^, a specific tRNA whose anticodon matches the UGA codon. tRNA^[Ser]Sec^ is first charged with serine by seryl-tRNA synthetase and then converted to selenocysteyl-tRNA^[Ser]Sec^ by Sec synthase (SelA). SelA uses selenophosphate as the Se donor, which is provided by selenophosphate synthetase (SelD). In eukaryotes and archaea, the biosynthesis of Sec proceeds by a similar process as in bacteria except that an intermediate, *O*-phosphoseryl-tRNA^[Ser]Sec^, is involved and it arises by *O*-phosphoseryl-tRNA^[Ser]Sec^ kinase, on seryl-tRNA^[Ser]Sec^. The eukaryotic/archaeal Sec synthase SecS then acts on *O*-phosphoseryl-tRNA^[Ser]Sec^ to generate Sec-tRNA^[Ser]Sec^ (Xu et al. 2007; Squires and Berry 2008; Allmang et al. 2009). In addition, as the SECIS element is localized in the 3′-untranslated region of selenoprotein mRNA in eukaryotes, and several additional proteins (such as a SECIS binding protein SBP2 and ribosome protein L30) are required for the incorporation of Sec into protein (Copeland and Driscoll 2001; Squires and Berry 2008).

In some prokaryotes, Se (in the form of selenophosphate) can be used for the biosynthesis of a modified tRNA nucleoside, 5-methylaminomethyl-2-selenouridine (mnm^5^Se^2^U, or SeU), which is located at the wobble position of the anticodons of some tRNAs (e.g., tRNA^Lys^, tRNA^Glu^, and tRNA^Gln^) (Ching et al. 1985; Wittwer and Ching 1989; Romero et al. 2005). The 2-selenouridine synthase (YbbB) is essential for the sulfur(S)-to-Se substitution in 2-thiouridine in these tRNAs (Wolfe et al. 2004). Besides, a third SelD-dependent Se utilization trait was reported in some bacteria, in which Se might be used as a Se-containing cofactor by certain molybdenum-containing hydroxylases, and two specific genes (YqeB and YqeC), whose function is unclear as yet, were predicted to be involved in this process (Haft and Self 2008; Zhang et al. 2008). Therefore, the whole Se utilization trait known to date is composed of three subsystems: Sec-decoding, SeU-utilizing, and Se-containing cofactor traits. Each trait has unique genes, whereas SelD is considered as a general signature for Se utilization (Zhang et al. 2008). A general scheme of the three known Se utilization traits in bacteria is shown in supplementary figure S1, Supplementary Material online.

As the Sec-decoding trait is the main biologic system of Se utilization, the majority of studies on Se focused on investigation of diversity and function of selenoproteins, most of which exhibit redox function. In recent years, with the rapid growth of genome sequencing projects and the fast-expanding application of bioinformatics approaches, the selenoproteomes of a variety of Sec-decoding organisms and environmental samples have been successfully identified (Kryukov et al. 2003; Zhang et al. 2005; Lobanov et al. 2007; Zhang and Gladyshev 2008b). For example, 25 and 24 selenoproteins have been reported in human and mouse, respectively (Kryukov et al. 2003). On the other hand, Se can be toxic at higher levels of accumulation in the cell (Zwolak and Zaporowska 2012). However, compared with other trace metals such as zinc and copper, the full details of Se metabolism and the associated regulatory network are not clear and the factors involved remain unidentified, which may include Se-specific transport system, chaperone for Se trafficking and Se-related transcriptional regulators.

Comparative methods have been developed to identify new genes involved in the metabolism of different trace elements including Se (Zhang and Gladyshev 2009, 2010, 2008a; Zhang et al. 2008, 2009). In this study, we used comparative genomics approaches to investigate all known Se metabolic pathways in more than 2,300 sequenced prokaryotic genomes. Occurrence of all known components involved in Se utilization (e.g., SelA, SelB, SelD and YbbB) could be easily identified, which revealed the largest utilization map of Se in prokaryotes thus far. Based on the genomic context information of these known genes, our study also generated evidence for new candidate genes involved in Se homeostasis in different organisms, including a predicted Se transporter, a Se-related transcriptional regulator, and a potential chaperone that is related to Se trafficking. Overall, these data provide new insights for better understanding of Se utilization and homeostasis. Moreover, our method could be easily extended to discover novel genes for many other processes.

## Materials and Methods

### Genomes, Sequences, and Other Resources

Sequenced bacterial and archaeal genomes from the Entrez Microbial Genome Database at National Center for Biotechnology Information (NCBI) were used in this study. Only one strain was selected from each species which has multiple sequenced strains (e.g., *Enterococcus faecalis V583* was used as a representative of *E. faecalis*). More than 2,100 bacterial and 215 archaeal genomes were analyzed (as of March 2013).

Based on our previous results, we used *E. coli* SelA, SelB, and SelD proteins as queries to search for components of the Sec-decoding trait, SelD and YbbB proteins for the SeU trait (Wolfe et al. 2004; Zhang et al. 2006) as well as SelD, YqeB (alternatively named HP1), and YqeC (alternatively named HP2) proteins for the third Se-containing cofactor trait (Zhang et al. 2008). For each of these proteins, TBLASTN (Altschul et al. 1990) was first used to identify genes coding for homologs with the cutoff of *E* value ≤0.1. Orthologous proteins were defined using the conserved domain database (COG, Pfam, or CDD) and bidirectional best hits (Tatusov et al. 2000; Wolf and Koonin 2012).

In addition, we selected ten genes upstream and downstream of the genomic region which contains all or most known genes involved in Se utilization in some organisms. For each of these proteins, similar computational approaches were used to verify their occurrence in different organisms. When necessary, orthologs were also confirmed by genomic location analysis or building phylogenetic trees for the corresponding protein families.

The STRING (Search Tool for the Retrieval of Interacting Genes/Proteins) database and programs (Franceschini et al. 2013) were also used to further identify gene candidates that may be functionally related to Se metabolism. Considering that some proteins may not have known COG domains, we used STRING with the protein-mode interactor to examine the functional linkages based on neighborhood, gene fusion and co-occurrence prediction methods. Only candidates with the confidence score greater than 0.4 were considered as possible candidates.

### Multiple Alignment and Phylogenetic Analysis

Multiple sequence alignments were performed using ClustalW (Thompson et al. 1994) and Mafft (Katoh and Toh 2008) with default parameters. Phylogenetic trees were reconstructed by PHYLIP programs (Felsenstein 1989). Pairwise distance matrices were calculated by PROTDIST to estimate the expected amino acid replacements per position. Neighbor-joining trees were obtained with NEIGHBOR and the most parsimonious trees were determined with PROTPARS. The robustness of the trees was evaluated by two additional algorithms, PHYML (Guindon and Gascuel 2003) and MrBayes (Ronquist and Huelsenbeck 2003).

## Results

### General Occurrence of SelD-Dependent Se Utilization Pathways in Prokaryotes

**Fig. 1.—:**
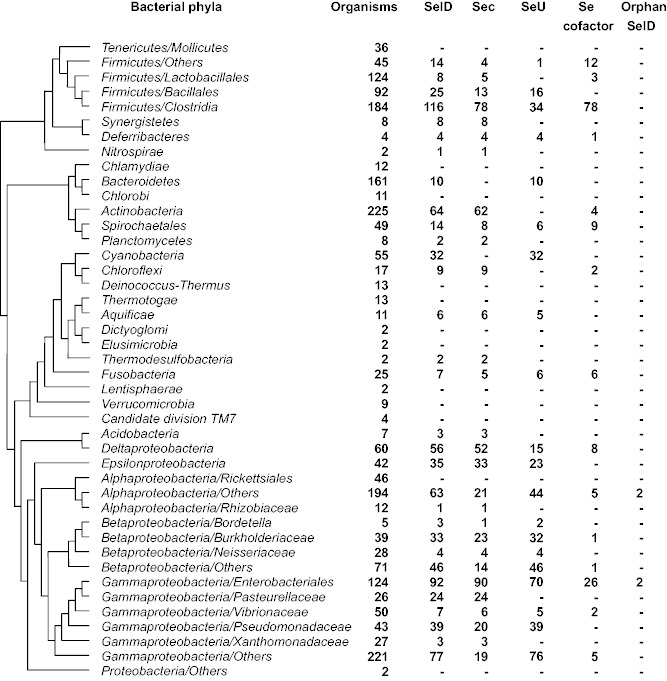
Occurrence of SelD and known Se utilization traits in bacteria. The tree is based on the bacterial part of a highly resolved phylogenetic tree of life (Ciccarelli et al. 2006). Phyla only containing single sequenced genome are not shown. Orphan SelD represents organisms that contain SelD but lack components of any of the known Se utilization traits.

Although it has been reported that the majority of prokaryotic organisms do not use Se (Zhang and Gladyshev 2009), sequence analysis of bacterial genomes revealed a wide distribution of genes encoding key components of Sec-decoding (SelA/SelB/SelD), SeU-utilizing (SelD/YbbB), and Se-containing cofactor (SelD/YqeB/YqeC) machinery. Figure 1 shows the distribution of the three Se metabolic pathways in different bacterial taxa based on a highly resolved phylogenetic tree of life (Ciccarelli et al. 2006). Details of the distribution of SelD and other genes involved in different Se utilization traits are shown in supplementary table S1, Supplementary Material online.

SelD is required for all three Se traits and has been defined as a general signature for Se utilization in biology (Zhang et al. 2008). Here, 812 SelD-containing organisms (38.3% of all sequenced bacteria) were identified. This gene was found to be present in nearly all bacterial phyla with the exception of a small number of clades such as Chlamydiae, Chlorobi, Mollicutes, Deinococcus-Thermus, and Thermotogae, which is consistent with our previous hypothesis that Se utilization is an ancient metabolic trait that was common to almost all species in this domain of life, and most organisms lost the ability to utilize Se (Zhang et al. 2006).

**Fig. 2.—:**
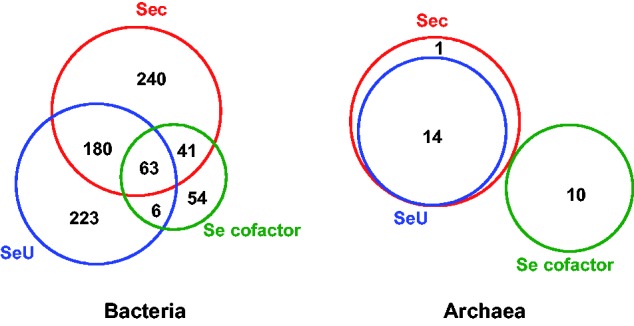
Distribution of known Se metabolic pathways in prokaryotes. Relationships among different Se utilization traits in archaea and bacteria are shown using Venn diagrams. The number of organisms utilizing corresponding Se pathways is indicated.

A total of 524 (24.7%), 472 (22.3%), and 164 (7.7%) organisms possess Sec-decoding, SeU-utilizing, and Se-containing cofactor traits, respectively (fig. 2). The majority of Sec-decoding organisms belong to Proteobacteria and Firmicutes (especially Beta-, Delta-, Epsilon-, Gamma-proteobacteria, and Firmicutes/Clostridia subdivisions), whereas Bacteroidetes and Cyanobacteria lack this trait. The SeU-utilizing trait was found to be completely absent in some phyla, which possessed the Sec-decoding trait (such as Actinobacteria, Synergistetes, Chloroflexi, and Gammaproteobacteria/Pasteurellaceae), but present in many Cyanobacteria and Bacteroidetes, all of which lack selenoproteins. The third Se-containing cofactor trait was mainly observed in different subdivisions of Firmicutes (such as Clostridia and Others) and Gammaproteobacteria/Enterobacteriales. Pearson’s Chi-square test suggested a significant relationship between any of the three Se utilization pathways (*P*-values for the relationship between Sec-decoding and SeU-utilizing traits, SeU-utilizing and Se-containing cofactor traits as well as Sec-decoding and Se-containing cofactor traits are 2.2e-16, 2.29e-9, and 1.61e-08, respectively; all Pearson’s Chi-square tests were calculated based on the total number of sequenced bacteria collected in this study). This is consistent with our previous assumption that one Se utilization trait may facilitate acquisition or maintenance of others due to the common gene SelD (Zhang et al. 2006). However, the occurrence of many organisms containing only one of these traits also suggests a relatively independent and complementary relationship among them.

More restricted utilization of Se was observed in archaea (fig. 2; supplementary table S2 and fig. S2, Supplementary Material online). Only 12.1% sequenced archaea contain SelD, which belong to four different orders of Euryarchaeota: Methanopyrales, Methanococcales, Halobacteriales, and Methanomicrobiales. Compared with bacteria, all SeU-utilizing organisms use Sec, implying a potential relationship between Sec and SeU utilization traits in archaea (*P*-value = 2.2e-16, Pearson’s Chi-square test). In contrast, all organisms using Se-containing cofactor belong to Halobacteriales which lack the Sec and SeU utilization, indicating that the Se-containing cofactor trait may not depend on the other two Se traits in this domain of life. Considering that only few organisms have SelD genes and that all SeU-utilizing archaea belong to Methanococcales, bias might be present when studying the interaction between different Se utilization traits in this domain.

Interestingly, at least four bacteria and one archaeon were found to have orphan SelD (containing SelD but lacking components of any of the known Se utilization trait) (supplementary table S1, Supplementary Material online). Such organisms included *Brevundimonas* sp. *BAL3* and *Methylobacterium nodulans* (Alphaproteobacteria/Others), *Xenorhabdus bovienii* and *Xenorhabdus nematophila* (Gammaproteobacteria/Enterobacteriales), and *Methanoplanus petrolearius* (the only SelD-containing organism in Euryarchaeota/Methanomicrobiales). It is possible that unknown SelD-related Se utilization pathway and genes may be present in these organisms. However, the possibility that these orphan SelD genes are either pseudogenes or involved in non-Se-related processes could not be neglected.

**Fig. 3.—:**
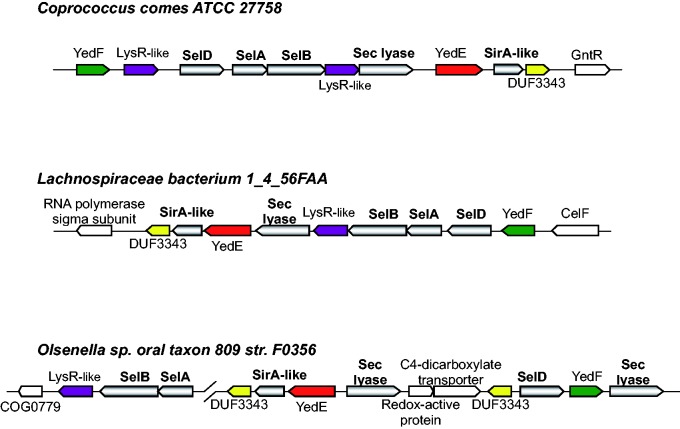
Genomic context of Se/Sec-related genes in representative genomes. Candidate genes involved in Se/Sec metabolism are shown in different colors. Coding direction is also indicated.

In several organisms, such as *Coprococcus comes*, *Lachnospiraceae bacterium 1_4_56FAA,* and *Slackia heliotrinireducens*, all known Sec-decoding genes and some known Se-related genes (e.g., Sec lyase and SirA-like protein) were found to be colocalized within one or two small genomic regions or even operons (fig. 3). Such regions contain additional genes which have never been reported to be associated with Se/Sec metabolism. It has been known that many clustered genes, especially those in the same operon, may be functionally related and/or coregulated (Brouwer et al. 2008). On the other hand, the sister species of some of these organisms lack either Sec utilization or any Se utilization pathway, indicating that a very recent horizontal gene transfer of the whole Sec/Se utilization trait might have happened in these organisms. Thus, new Se-related genes might be present in these gene clusters.

### Identification of New Candidate Genes Involved in Se Metabolism

To identify new Se-related genes, we examined ten genes upstream and downstream of selected genomic regions which contain all or most known genes involved in Se utilization in these organisms. Based on the occurrence, genomic location, and phylogenetic analyses of these genes, four candidate genes (highlighted in fig. 3) were chosen for further analysis.

#### YedE

The *YedE* gene (TIGR04112) is often found to be located next to known Se/Sec-related genes such as SirA-like, SelD, and Sec lyase in many organisms in different bacterial phyla, implying that this protein may be involved in Se metabolism. We also used the STRING database to examine proteins that might be functionally linked to YedE based on genomic context, gene fusion, and co-occurrence analyses (table 1, using *S. heliotrinireducens* as representative organism). Consistent with our observation, the proteins with the best score were SirA-like and Sec lyase. Thus, it appears that YedE may be functionally related to Se metabolism in spite that the specific function of YedE protein is not clear.

**Table 1:** STRING Analysis of Genes Functionally Associated with YedE, YedF, and DUF3343 in *Slackia heliotrinireducens*

Rank	YedE	YedF	LysR-like	DUF3343
	(Shel_25130)	(Shel_25450)	(Shel_25320)	(Shel_25430)
1	Shel_25120 (SirA-like)	Shel_25440 (Sec lyase family)	Shel_25330 (2-hydroxyglutaryl-CoA dehydratase subunit)	Shel_25440 (Sec lyase family)
2	Shel_25440 (Sec lyase family)	Shel_25430 (DUF3343)	Shel_25310 (SelA)	Shel_25450 (YedF)
3	Shel_25420 (CoA-substrate-specific enzyme activase)	Shel_25420 (CoA-substrate-specific enzyme activase)	Shel_25300 (SelB)	Shel_25420 (CoA-substrate-specific enzyme activase)
4	Shel_25450 (YedF)	Shel_25360 (SelD)	Shel_23510 (methylenetetrahydrofolate reductase)	Shel_25460 (DUF3343)
5	Shel_25110 (DUF3343)	Shel_25120 (SirA-like)	Shel_23580 (1-deoxy-d-xylulose 5-phosphate reductoisomerase)	Shel_25360 (SelD)
6	Shel_25140 (glycosyltransferase)	Shel_04340 (transglutaminase-like enzyme)	—	—
7	Shel_07530 (DUF169)	Shel_25470 (2-hydroxyglutaryl-CoA dehydratase subunit)	—	—
8	—	Shel_25460 (DUF3343)	—	—
9	—	Shel_09640 (ribosomal large subunit pseudouridine synthase B)	—	—
10	—	Shel_06730 (aspartyl aminopeptidase)	—	—

To investigate the possible function of YedE, we first analyzed the distribution of this protein family in all sequenced bacteria. Orthologs of YedE were detected in a variety of organisms from different bacteria phyla (Bacteriodes, Firmicutes, Spirochetes, Synergistetes, delta-proteobacteria, epsilon-proteobacteria, etc), almost all of which contain SelD (supplementary table S1, Supplementary Material online). Pearson’s Chi-square test revealed a significant relationship between YedE and SelD-related Se utilization trait (*P*-value = 2.2e-16). Using TMHMM software (Sonnhammer et al. 1998), we found that YedE is a membrane protein which typically contains ten transmembrane regions. Similar relationship between YedE and SelD was also observed in archaea (supplementary table S2, Supplementary Material online). Surprisingly, the YedE gene is split into two neighboring genes in archaea, each of which contains 4–5 transmembrane regions.

Interestingly, we found that YedE proteins are homologous to some proteins in several organisms such as *Serratia* species, which are thought to function in the transport of S-containing molecules (Gristwood et al. 2011). These S-related transporters contain several conserved glycines and an invariant cysteine (Cys) that is probably an important functional residue. Multiple alignment of YedE proteins and its S-related homologs suggested that all YedE proteins have the same functionally conserved Cys (supplementary fig. S3, Supplementary Material online). In addition, YedE contains specific residues including additional Cys residues, some of which might be related to the specific function of YedE. Considering that Se and S are closely related elements that exhibit similar chemical properties, YedE may either have evolved from or share a common ancestor with its S-related homologs. A reasonable hypothesis is that YedE is most likely involved in the transport of Se-containing molecules.

#### YedF

The second candidate gene is *YedF* (TIGR03527), whose protein contains two redox-related domains: The N-terminal SirA-like domain (pfam01206) and the C-terminal DsrE-like domain (pfam02635). This gene (previously named SirA-like) has been suggested to be associated with SelD based on our previous analysis (Zhang et al. 2008).

STRING analysis of YedF suggested that this protein may be functionally related to several Se-related proteins including Sec lyase and SelD (table 1). Comparative analysis of YedF proteins revealed that this family is present in a broad taxonomic range of bacteria (supplementary table S1, Supplementary Material online) but is absent in archaea. Almost all YedF-containing organisms have SelD, implying a strong linkage between YedF and SelD-related Se metabolism (*P*-value = 2.2e-16, Pearson’s Chi-square test). However, a small number of sequenced organisms that lack SelD (such as *Coriobacterium glomerans* and *Clostridium beijerinckii*) also have YedF, indicating that it may be involved in non-SelD-related processes in these organisms.

Although the function of YedF is unknown, both domains within this protein contain highly conserved functional motifs (supplementary fig. S4, Supplementary Material online). The SirA-like domain contains a CPXP (X represents any amino acid) motif which may play a significant structural role in stabilizing the first helix as part of the new type N-capping box in some proteins (Katoh et al. 2000). On the other hand, the DsrE-like domain has a typical redox-active motif CGXC, which might participate in intracellular S reduction in some bacteria (Pott and Dahl 1998). Thus, it is possible that members of YedF are involved in Se-related redox processes. Further experiments will be needed to verify its function.

#### LysR-Like

A *LysR-like* gene was also found to be next to *SelA*/*SelB*/*SelD* genes in several evolutionarily distant organisms, which was further supported by STRING analysis (table 1). These proteins belong to a large LysR transcriptional regulator superfamily (COG0583). Members of this superfamily have an N-terminal DNA-binding domain and a C-terminal substrate-binding domain (Henikoff et al. 1988). The topology of this substrate-binding domain is most similar to that of the type 2 periplasmic binding proteins, which are responsible for the uptake of a variety of substrates such as phosphate, sulfate, polysaccharides, lysine/arginine/ornithine, and histidine (Henikoff et al. 1988).

**Fig. 4.—:**
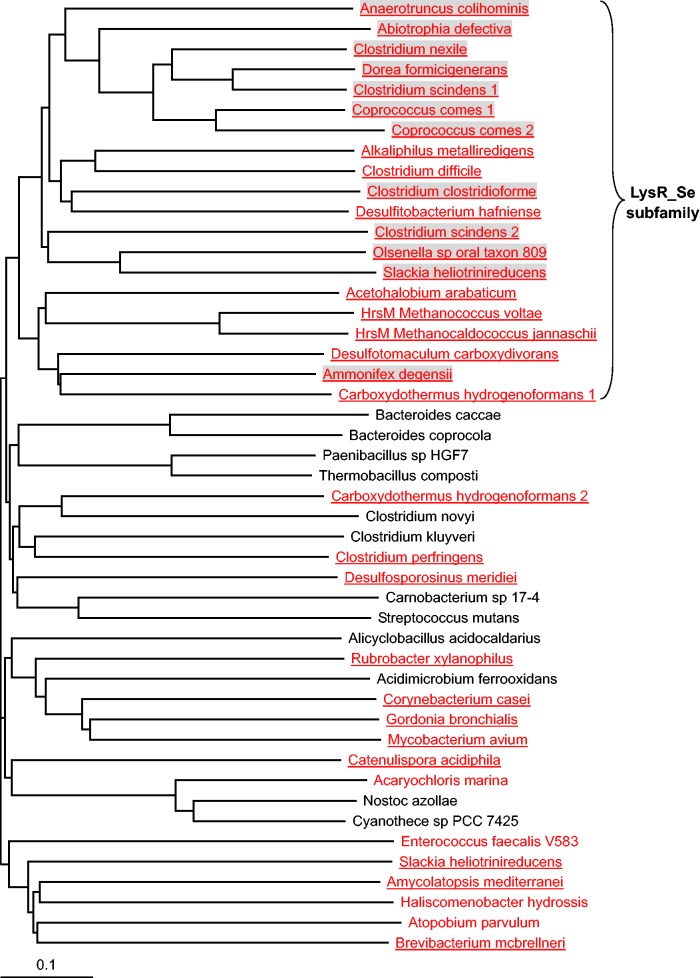
Phylogenetic analysis of LysR-like proteins. Representative SelD-containing and SelD-lacking organisms are shown in red and black, respectively. Sec-decoding organisms are shown in red and underlined. Organisms in which LysR-like gene is very close to Sec-decoding genes are shaded. The LysR_Se subfamily is indicated.

To investigate the relationship between LysR-like protein and SelD-dependent Se utilization, we examined the distribution of LysR-like protein and found that its homologs were widespread in prokaryotes and all LysR-containing organisms have multiple members of this superfamily. For example, *E. coli* has more than 20 LysR-like genes. Considering that different proteins of LysR superfamily may have different functions and that some of these genes are located very close to Sec-decoding genes in some organisms, we therefore hypothesized that there might be a Se-related LysR-like subfamily. Phylogenetic analysis of LysR-like proteins from sequenced bacteria and archaea revealed that a new LysR-like subfamily (named LysR_Se thereafter) might be functionally linked to Se/Sec metabolic pathways (fig. 4). Orthologs of LysR_Se proteins (also named HrsM in archaea) were only detected in SelD-containing organisms almost all of which have Sec-decoding trait. In addition, all LysR-like proteins whose genes are located close to Sec-decoding genes belong to LysR_Se subfamily. These observations suggested a strong correlation between LysR_Se and Sec biosynthesis (*P*-value < 2e-16, using Pearson’s Chi-square test).

Although significant similarity was observed between sequences in this putative LysR_Se subfamily and other LysR homologs, multiple alignment of LysR_Se sequences suggested specific residues which are only present in the Se-related subfamily, especially a highly conserved proline residue in the substrate-binding domain, which might play a key role in substrate recognition (supplementary fig. S5, Supplementary Material online).

#### DUF3343

In some SelD-containing organisms, a gene encoding a small protein (Pfam11823, DUF3343, typically 80–100 amino acids) is located very close to the YedF or Sec lyase genes. STRING analysis also implied a strong functional link between DUF3343 and Sec lyase/YedF (table 1). However, the function of DUF3343 proteins has not been characterized yet.

**Fig. 5.—:**
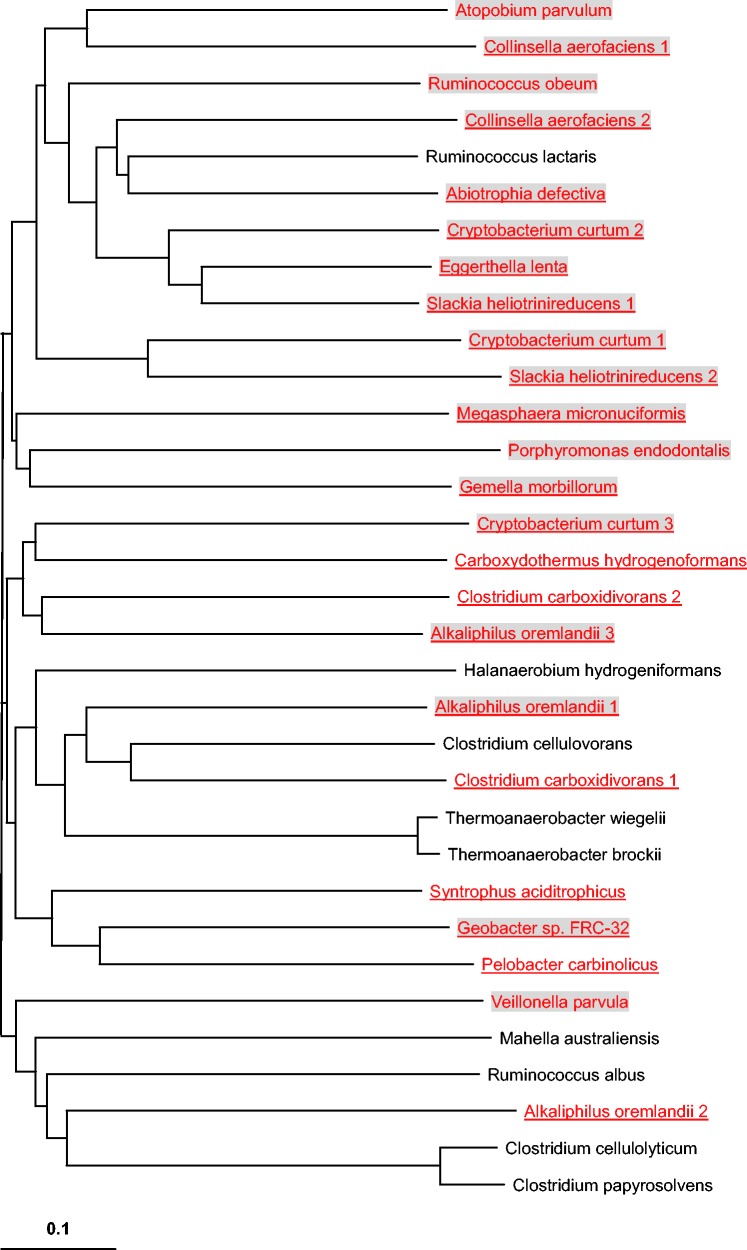
Phylogenetic analysis of DUF3343 proteins. SelD-containing and SelD-lacking organisms are shown in red and black, respectively. Sec-decoding organisms are shown in red and underlined. Organisms in which DUF3343 gene is very close to other genes involved in Se metabolism are shaded.

Homologs of DUF3343 were detected in many organisms in the majority of bacterial and archaeal phyla, including both SelD-containing and SelD-lacking organisms. Moreover, many organisms contain multiple DUF3343 homologs. Similar to the analysis of LysR-like protein, we tried to identify a Se-related DUF3343 subfamily. However, no such clear branch could be found. Figure 5 shows the phylogenetic tree of DUF3343 proteins from representative organisms. A highly conserved Cys residue was observed in DUF3343 sequences from both SelD-containing and SelD-lacking organisms, implying an important role in the general function of different DUF3343 proteins (supplementary fig. S6, Supplementary Material online).

## Discussion

In the past decade, much effort has been devoted to identifying Sec insertion machinery and new selenoprotein genes (Kryukov et al. 2003; Zhang et al. 2005, 2008; Lobanov et al. 2007; Zhang and Gladyshev 2008b). To date, three SelD-dependent Se utilization traits have been successfully identified, all of which use selenophosphate as the Se donor. In this project, we extended previous studies to identify novel candidate genes that may participate in Se metabolism by including much more recently sequenced genomes. To our knowledge, these data represent the most comprehensive analysis of genes that are known or likely to be involved in Se utilization in sequenced prokaryotes.

By using comparative genomics approaches, we analyzed the distribution of all known Se utilization traits as well as related genes in both bacteria and archaea. The widespread taxa distribution of Se utilization traits is consistent with the idea that Se could be used by a variety of organisms in almost all bacterial phyla. However, that fact that less than 40% of sequenced organisms contain at least one Se utilization trait revealed that the majority of species lost the ability to use this trace element. Although it is unclear whether one Se utilization pathway may influence the evolution of the other two, significant overlaps between them suggested that they might have evolved under similar environmental conditions in many organisms.

Several previous studies have shown that many marine or aquatic bacteria possess Sec-decoding trait (Zhang et al. 2005; Zhang and Gladyshev 2008b, 2010). However, the number of sequenced organisms examined in these studies was limited. Here, we analyzed living environments of all sequenced bacterial species (supplementary table S1, Supplementary Material online). Approximately half of marine/aquatic bacteria (45.2%, 224 genomes) contain at least one Se utilization trait. On the other hand, only one-third of nonaquatic bacteria (36.4%, 588 genomes) could use Se. Thus, our results are basically consistent with previous observation that marine or aquatic bacteria appear to be more likely to use Se. Considering that the number of sequenced aquatic bacteria is much smaller than that of nonaquatic bacteria, future studies are needed with the increasing of new aquatic bacterial genomes.

Comparative and phylogenetic analyses of the genomic context of known Se-related genes suggested new candidate genes that might be involved in Se metabolism in prokaryotes, including genes encoding YedE, YedF, LysR_Se, and DUF3343 proteins. However, significant homologs of these proteins could not be detected in eukaryotes, implying that these genes might be associated with Se utilization exclusively in prokaryotes.

YedE protein is predicted as a membrane protein and is distantly similar to S-related transporters. In addition, all YedE proteins contain both functionally important residues found in its S-related homologs and several unique residues. Although it has been reported that uptake of selenate occurs mainly through the sulfate transport system (Aguilar-Barajas et al. 2011), it is possible that YedE evolved from or shared a common ancestor with certain S-related transporters and has raised new function involved in transport of Se-containing molecules, which could be distinguished by some of those YedE-specific residues. As the sulfate transporter is known to be responsible for the uptake and accumulation of Se in the cell, an attractive hypothesis is that YedE might serve as a Se exporter that mediates export of excess Se.

YedF protein contains both SirA-like and DsrE-like domains. Homologs of SirA-like proteins are widespread in almost all bacteria, which belong to a large superfamily and may have different functions. Previously, we could not find a SirA-like subfamily that may be associated with Se utilization (Zhang et al. 2008). However, the co-occurrence of both SirA-like and DsrE-like domains in YedF proteins could be considered as a signature for identification of YedF orthologs. The CGXC redox motif in the DsrE-like domain of YedF protein implied that this protein might play a role in Se-related redox processes (say, Se detoxification) by the mechanism similar to S reduction. Very recently, it was reported that YedF (alternatively named FdhU) may regulate Se-dependent biogenesis and expression of formate dehydrogenase in *Campylobacter jejuni*, which may provide additional evidence for the relationship between YedF and Se utilization (Pryjma et al. 2012; Shaw et al. 2012).

A new LysR_Se subfamily that belongs to a large LysR-type transcriptional regulator superfamily was also identified in a variety of organisms all of which contain Se utilization traits (especially Sec-decoding trait). One important LysR subfamily is CysL, a regulator of S metabolism, which activates the transcription of the *cysJI* operon encoding sulfite reductase in bacteria (Guillouard et al. 2002). In *E. coli*, three LysR-type proteins are known to be involved in the regulation of S metabolism: CysB, Cbl, and MetR (Gyaneshwar et al. 2005; Stec et al. 2006). Recent studies demonstrated that the HrsM protein (LysR_Se ortholog in archaea) can repress the transcription of Se-free (Cys-containing) hydrogenases (Frc and Vhc operons) in Sec-decoding archaea *Methanococcus* species when Se is available (Sun and Klein 2004; Hohn et al. 2011). It was also suggested that selenophosphate might be a likely effector for the activation of HrsM (Hohn et al. 2011). Very recently, it was also found that disruption of HrsM gene in *Methanococcus maripaludis* may result in a dramatically downregulation of both selenoproteins and its Cys-containing homologs, suggesting its involvement in regulation of both Sec- and Cys-encoding isogenes (Fersch et al. 2013). Overall, these studies implied that LysR_Se subfamily might have evolved from an S-related LysR ancestor and serve as a key transcriptional regulator of genes related to Se metabolism (especially Sec biosynthesis genes), most likely by sensing the intracellular amount of selenophosphate, the product of SelD.

The DUF3343 protein was also predicted to be associated with Se metabolism by comparative genomics analysis. Although its function is unclear, we hypothesized that DUF3343 might be related to Se-containing molecule trafficking within the cell. First, the size of DUF3343 is comparable with some metal chaperones, such as CopZ for copper and metallothionein for heavy metals. Second, YedF and Sec lyase, the two genes which DUF3343 is often clustered with, are putatively involved in Se utilization and recycling, especially Sec lyase that supplies Se (in the form of selenide) for selenoprotein biosynthesis through decomposition of Sec (Zhang et al. 2008; Kurokawa et al. 2011). During these processes, a potential mechanism might be needed to deliver Se compound to its targets and DUF3343 might be the key component. On the other hand, the lack of a Se-specific DUF3343 subfamily implied that DUF3343 proteins may have Se-independent function. Thus, this small protein family might be responsible for the intracellular trafficking of either Se or S molecules in different organisms, which is similar to metallothionein that may bind a wide range of heavy metals (Vasak 2005).

**Fig. 6.—:**
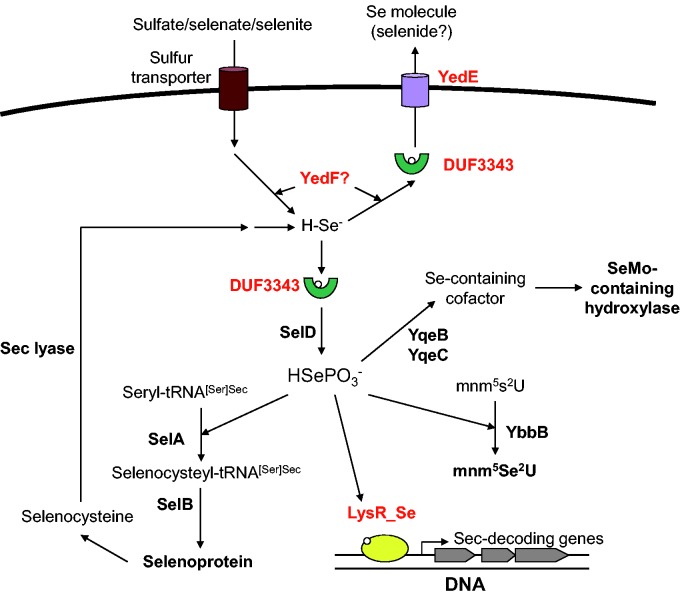
A proposed model for Se homeostasis in bacteria. Known Se/Sec-related genes are shown in black, whereas predicted genes are highlighted in red.

Comparative genomics analysis of Se utilization may offer an opportunity to identify new genes involved in the Se metabolism and associated regulatory network. Based on the data described above, we proposed a general model for Se homeostasis in bacteria (fig. 6). It should be noted that the four genes predicted in this study only co-occurred in a limited number of organisms, and many other Se-utilizing organisms only possess some or even lack all of them. Thus, additional genes and/or unspecific mechanisms might be involved in maintaining Se homeostasis in some of these organisms. In addition, the possibility that some of these genes are involved in S metabolism in some organisms could not be excluded. In the future, experimental studies are needed to validate the specific function of these genes.

## Conclusions

In this study, we carried out comparative genomics studies to search for novel genes which are strongly linked to Se metabolism and homeostasis in prokaryotes. Our data highlight a complex and dynamic evolutionary process for all known Se utilization traits. Several candidate genes were found to be involved in Se metabolism in different species, including a predicted Se-related transporter YedE, a redox protein YedF, a Se-specific transcriptional regulator LysR_Se that may mainly regulate the expression of Sec-decoding genes, and a possible chaperone DUF3343 protein involved in Se or S trafficking in the cell. Multiple sequence alignment suggested conserved functional residues for some of these protein families. Finally, a general model for Se metabolism in bacteria was proposed, which might help understand the details of the complete metabolic network of this essential micronutrient in biology.

## Ethics

This study does not require ethical approval (granted by the Ethics Committee of Institute for Nutritional Sciences, Shanghai Institutes for Biological Sciences, Chinese Academy of Sciences).

## Supplementary Material

Supplementary figures S1–S6 and tables S1 and S2 are available at *Genome Biology and Evolution* online (http://www.gbe.oxfordjournals.org/).
